# Determination and Prediction of Respirable Dust and Crystalline-Free Silica in the Taiwanese Foundry Industry

**DOI:** 10.3390/ijerph15102105

**Published:** 2018-09-25

**Authors:** Ching-Tang Kuo, Fen-Fen Chiu, Bo-Ying Bao, Ta-Yuan Chang

**Affiliations:** 1 Department of Public Health, College of Public Health, China Medical University, No. 91, Hsueh-Shih Road, Taichung 40402, Taiwan; ctkuo228@gmail.com; 2 Department of Occupational Safety and Health, College of Public Health, China Medical University, No. 91, Hsueh-Shih Road, Taichung 40402, Taiwan; jessica.cy@msa.hinet.net; 3 Department of Pharmacy, College of Pharmacy, China Medical University, No. 91, Hsueh-Shih Road, Taichung 40402, Taiwan; bao@mail.cmu.edu.tw; 4 Department of Nursing, Asia University, No. 500, Lioufeng Road, Wufeng, Taichung 41354, Taiwan

**Keywords:** crystalline silica, exposure assessment, foundry industry, predictive model, respirable dust

## Abstract

*Background:* Respirable crystalline silica (RCS) has been recognized as a human carcinogen; however, the measurement and analysis of RCS in small-scale foundries is rare and difficult. This study aimed to measure respirable dust and RCS levels among 236 foundry workers in Taiwan and used these data to establish predictive models for personal exposure. *Methods:* Personal sampling of various production processes were measured gravimetrically and analyzed using the X-ray diffraction method. Multiple linear regression was used to establish predictive models. *Results:* Foundry workers were exposed to geometric means and geometric standard deviations of 0.52 ± 4.0 mg/m^3^ and 0.027 ± 15 mg/m^3^ for respirable dust and RCS, respectively. The highest exposure levels were observed among workers in the sand blasting process, with geometric means of 1.6 mg/m^3^ and 0.099 mg/m^3^ for respirable dust and RCS, respectively. The predictive exposure model for respirable dust fitted the data well (R^2^ = 0.75; adjusted R^2^ = 0.64), and the predictive capacity for RCS was higher (R^2^ = 0.89; adjusted R^2^ = 0.84). *Conclusions:* Foundry workers in the sand blasting process may be exposed to the highest levels of respirable dust and RCS. The developed models can be applied to predict respirable dust and RCS levels adequately in small-scale foundry workers for epidemiological studies.

## 1. Introduction

Many studies have reported on the association between exposure to respirable crystalline silica (RCS) and adverse health effects, including silicosis, lung cancer, non-malignant respiratory disease, and possibly kidney disease [1,2,3,4,5]. The International Agency for Research on Cancer (IARC) reviewed the scientific literature and designated crystalline silica (i.e., quartz, cristobalite, and tridymite) as a Group 1 human carcinogen [6,7,8]. Moreover, in the United States, the National Institute for Occupational Safety and Health (NIOSH) found a 30% increase in the incidence of lung cancer due to crystalline silica exposure [9].

Many organizations have established occupational exposure limits for crystalline silica, but the standard varies across agencies. The Occupational Safety and Health Administration (OSHA) in the United States recently established a new permissible exposure limit (PEL) of 50 μg/m^3^ to reduce the risk of silicosis, lung cancer, other lung diseases, and kidney disease [10,11]. This exposure limit has been recommended by the NIOSH since 1978 [12]. However, the lower exposure limit of 25 μg/m^3^ was proposed by the American Conference of Governmental Industrial Hygienists (ACGIH) for an average over an eight-hour day in 2009 [13]. In Europe, a binding occupational limit of 0.10 mg/m^3^ for process generated RCS was agreed to by industry and the European Commission Advisory Committee for Safety and Health at work (ACSH) in 2013 [14]. In Taiwan, the dust has been classified into four categories, including type I dust (i.e., dust with the crystalline silica ≥10%), type II dust (i.e., dust containing crystalline <10%), type III dust (defined for asbestos) and type IV dust (i.e., nuisance dust). The PEL of type I dust is 10 mg/m^3^ divided by 2 and the percentage of crystalline silica that generates PELs of 0.83 mg/m^3^ (i.e., 10/[2 + 10]) and 0.10 mg/m^3^ (i.e., 10/[2 + 98]) for 10% and 98% crystalline silica, respectively. Conversely, the PEL of type II dust is 1 mg/m^3^ and 5 mg/m^3^ for nuisance dust [15].

Extensive research has shown that foundry industry workers have been exposed to high levels of silica dust [16,17,18,19]; however, the human health risks at small-scale companies with serious exposure and poor resources have not been investigated [20]. Occupational epidemiological studies have observed significantly higher rates of morbidity and mortality for lung cancer among foundry workers across East Asia [21,22], Europe [23,24], and the United States [25]. An increased prevalence of pneumoconiosis was reported among foundry workers in comparison to administrative workers in Taiwan [26]. Respirable dust and total dust are common in such workplaces; however, the proportion of crystalline silica is difficult to analyze because of limited budgets for environmental surveys and monitoring. Hence, a predictive model to estimate the potential concentration of RCS based on accessible measurements and information would be a cost-efficient and useful tool in epidemiological studies. Consequently, the purpose of this study is to measure the concentration of respirable dust and RCS among foundry industry workers in Taiwan and to establish a predictive model for RCS concentrations.

## 2. Materials and Methods

### 2.1. Study Population

**Figure 1:**
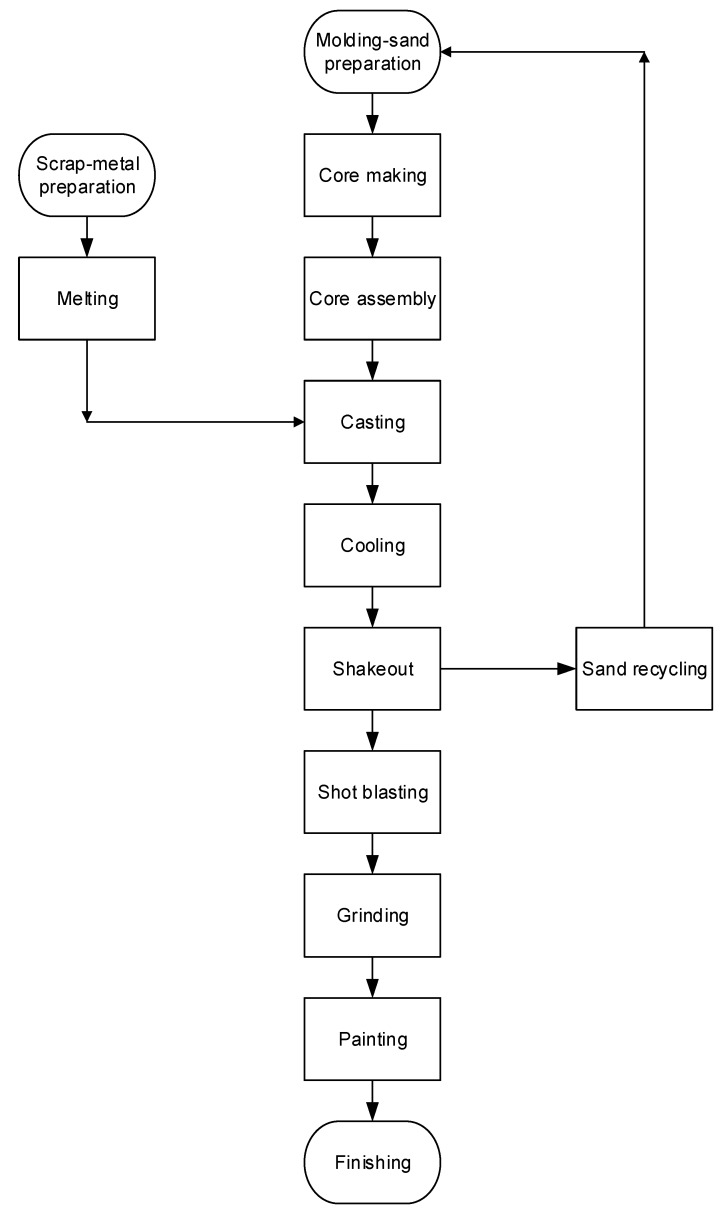
Main manufacturing processes of the foundry industry.

A list of potential candidate companies for RCS monitoring was obtained from the Taiwan Association of Casting Industry (TACI) in 2014. Data on the main products manufactured, employee numbers, geographic locations, and manufacturing processes were collected from the TACI website [27]. Three companies were selected as representative: an organization manufacturing large items which followed a mandated occupational safety and health program (Factory A; 78 workers); an organization manufacturing large items which did not follow the mandated occupational safety and health program (Factory B; 110 workers); and an organization manufacturing small items which did not follow a health and safety program (Factory C; 48 workers). A fourth company, manufacturing large products which did not follow the occupational safety and health program (Factory D; 88 workers), was used to test the validity of the predictive models established using data from Factories A–C. The selected factories produced various types of cast iron (i.e., nodular, grey, and ductile), and conducted machine-tool casting, Meehanite casting, general machine casting, and steel casting from different materials (e.g., carbon, friction-resisting, anti-corrosion, heat-resisting, stainless, and nickel-base). The main processes of the foundry industry are shown in Figure 1. Because this study is part of an exposure assessment in an epidemiological study, it has been reviewed and approved by the Institute Review Board of College of Public Health of the China Medical University (Ethic code: 100-03-10-04) before the implementation. Written informed consent was acquired from each company and participant before the commencement of the study.

### 2.2. Exposure Assessment and Sampling Strategy

A walk-through survey was conducted at each factory by an industrial hygienist, who collected information including the safety data sheet of used silica sand, details regarding production processes, the number of production-line workers, operating styles, casting materials and fuel, use of personal protective equipment, and climatic conditions (i.e., air-velocity direction, wind speed, temperature, relative humidity, and ventilation) in the workplace. Workers mixed silica sand with binders manually to prepare the mold and cores or collected scrap metal for melting in the furnace at 700–1200 °C before casting. After pouring the molten metal into a mold cavity with refractory cores to create void spaces, the mold was then moved for cooling. Shake out was conducted to remove the molding media around the metal casting and shot blasting was then performed. The remaining molding sand was recycled and conditioned for molding-sand preparation. Finally, grinding/fettling and painting completed the process.

Because each worker was responsible for at least two operations, subjects in each company were classified into one of six similar exposure groups (SEGs) based on their operating processes, tasks, and work locations as determined by the walk-through survey. These SEGs were modeling (i.e., molding-sand preparation, core making, and core assembly), casting (such as scrap-metal preparation, melting, casting, and cooling), sand-box cleaning (i.e., shakeout and sand recycling), sand blasting (i.e., shot blasting and transportation), polishing (such as grinding/fettling and painting), and office areas. Based on production scale, process characteristics, and employee numbers at each factory, one worker was selected to represent the similar exposure level for an average of eight workers in the same SEG. A total of 38 personal air samples were collected from the individual breathing zones of subjects among the four companies. Personal air sampling was performed for each worker with a shift from 09:00–16:00 (with a one-hour break taken for lunch) on work days during 2014–2015. Air samples obtained from office workers in each company were considered to be the reference group. In this study, there were 11 air samples from Factory A on December 16 (Tuesday) in 2014, 13 from Factory B on January 8 (Thursday) in 2015, six from Factory C on February 4 (Wednesday) in 2015, and eight from Factory D on March 25 (Wednesday) in 2015.

During personal air sampling, the wind speed, temperature, and relative humidity were measured simultaneously using an air-velocity meter (Velocicalc Model 9545, TSI Inc., Shoreview, MN, USA). The measured ranges (accuracy) for these atmospheric parameters were 0 to 30 m/s (±0.015 m/s), −10 to 60 °C (±0.3 °C), and 0 to 95% (±3%) for wind speed, temperature, and relative humidity, respectively. The instrument was calibrated before use.

### 2.3. Respirable Dust Sampling and Respirable Crystalline Silica Analysis

Personal air samples of respirable dust were obtained using the method established by the Institute of Labor, Occupational Safety and Health (ILOSH), Ministry of Labor, Taiwan (method number: 4003) [28]. A personal air sampling pump (AirChek 52, SKC Inc., Eighty Four, PA, USA) was set to an air flow rate of 2.5 L/min and linked to a 25 mm aluminum cyclone assembly (SKC225-01-01, SKC Inc., Eighty Four, PA, USA) with a 25 mm filter cassette that contained a glass microfiber filter coating with a polytetrafluoroethylene filter (T60A20, PALL Life Sciences, Westborough, MA, USA) for collecting air samples. The filter was analyzed gravimetrically using an electronic balance (ER-182A, A & D Mercury Ltd., Thebarton, Australia; accuracy: 0.01 mg) with conditioning at 25 ± 3 °C and 50 ± 5% relative humidity for 24 h before analysis. The cyclone assembly and air sampling pump were placed on each participant to collect respirable dust in their personal breathing zone during a six-hour shift. Because the workers did not wear personal protective respirators, the collected air samples present the personal external exposure in the workplace. These samples were then properly packaged and shipped to the laboratory for gravimetric and component analysis.

After the gravimetric analysis of respirable dust, the air sample was further analyzed for the concentration of respirable crystalline-free silica using an X-ray diffractometer (Shimadzu 6000, Shimadzu Corp., Kyoto, Japan) with an irradiation of Cu Kα (λ = 1.5418 Å, 40.0 kV, 30.0 mA). The instrument recorded from 5° to 70° (2θ) with a scanning step of 0.5°/min for qualitative measurements, and quartz, cristobalite, and tridymite were set at 26.2–27.0°, 21.4–22.4°, and 20.3–21.1° of 0.02°/min, respectively, for quantitative determination. A calibration curve was established for each composition that ranged from 0.03–5.16 mg/sample for quartz, 0.03–4.56 mg/sample for cristobalite, and 0.041–2.49 mg/sample for tridymite. All calibration curves had R^2^ ≥ 0.995. Background concentration was adjusted according to the Japanese Industrial Standards (JIS) method A1481 applied to the correction of aluminum-based substrate absorption [29]. The qualitative limit of detection (LOD) for quartz, cristobalite, and tridymite was 8 μg, 6 μg, and 42 μg, respectively. A reagent blank was analyzed after every 10 samples for quality assurance purposes to determine whether the analytical system was contaminated. If the blank value was higher than twice the LOD, the source of contamination or interference was identified and excluded before the re-analysis of the samples. Further laboratory details are available from the ILOSH [28]. For measured RCS below the LOD, the half LOD (i.e., 1.5 μg/m^3^) was recorded due to the highly skewed distribution of raw RCS data [30].

### 2.4. Statistical Analysis

The Shapiro—Wilk test was used to evaluate the normality of continuous variables, including respirable dust level, RCS level, wind speed, temperature, and relative humidity. Because these variables were not normally distributed, the non-parametric Kruskal–Wallis test was conducted to compare the differences between companies and/or processes. Later, the Wilcoxon rank sum test was used to perform the post-hoc examination for those variables with significant between-group differences. In addition, the median, range, geometric mean (GM), and geometric standard deviation (GSD) were calculated as the statistical descriptors.

The concentrations of respirable dust and RCS were used a base-10 logarithmic transformation to produce a normal distribution for further analysis. Simple linear regression was applied to identify the significant predictive variables for the normally distributed levels of respirable dust and RCS. Of the 27 RCS samples collected, 10 were less than the LOD (i.e., 0.003 mg/m^3^); hence, they were not included in the dataset to develop the predictive model to avoid interference and improve predictive capacity. Three personal samples collected from office workers at each company were used as the model reference group. Labor scale was defined as large (≥100 employees) or small (<100 employees) for companies. Both labor scale (as compared to the small company) and process variables (as compared to the office area) were coded as 1/0 in the analyses. Multiple linear regression models were performed to establish the predictive levels of respirable dust and RCS. Only those variables that produced a change greater than 10% in the adjusted R^2^ values for the base-10 logarithmically transformed levels of respirable dust and RCS were used in the final model [31,32]. A stepwise approach using Akaike Information Criteria (AIC) was applied to select variables in the final model, where the model with the lowest AIC value was preferred. All variables in the final model had *p* values < 0.10. A maximum variance inflation factor (VIF) of 10 was selected as the cutoff value to indicate excessive multi-collinearity between predictive variables [33]. The Durbin–Watson (DW) value was used to present a lack of autocorrelation in the residuals of a regression model as being higher than the test bound based on the significance level, sample size, and the number of included variables [34]. Furthermore, residual diagnostics were performed to test whether all the linear regression model assumptions were fitted in the analysis. The Statistical Analysis Software (SAS), standard package version 9.4 for Windows, was used for statistical analysis (SAS Institute Incorporation, Cary, NC, USA) and a significance level of 0.050 was used for all tests.

Finally, the measured respiratory dust and RCS data from Factory D was used to test the validity of the predictive model established using the data from three factories. We applied the following Equation [35]:

(1) $$\mathrm{accuracy}=\sqrt{{\mathrm{bias}}^{2}+{\mathrm{precision}}^{2}}$$

to calculate model accuracy using the sum of the square of the mean difference between the predictive and measured values (bias) and the square of the standard deviation of the mean difference (precision).

## 3. Results

The percentage of crystalline-free silica in dust samples ranged from non-detectable to 53.3%. Table 1 shows the measured levels of respirable dust and RCS as well as climatic variables in the workplace, by factory. Large variations in GM and GSD values of respirable dust (0.52 ± 4.0 mg/m^3^) and RCS (0.027 ± 15 mg/m^3^) were identified among foundry industry workers, and the highest GM values of respirable dust and RCS were found both in Factory B (0.83 mg/m^3^; 0.092 mg/m^3^), although no significant differences were observed between factories. Factories A and C had significantly higher temperatures as compared to those of Factory B. In addition, Factory A showed significantly higher relative humidity as compared to Factory B.

Personal exposure to respirable dust and RCS, and climatic conditions for the different SEGs are shown in Table 2. Only respirable dust levels and wind speed showed significant differences between the various processes. Sand blasting workers had the highest GM of respirable dust (1.6 mg/m^3^), and polishing workers had the highest median of wind speed (1.1 m/s), respectively. Workers in all processes except for sand-box cleaning were exposed to significantly higher medians of respirable dust levels and wind speed as compared to office workers. The highest RCS level (GM: 0.099 mg/m^3^; range: 0.0015–21 mg/m^3^) was identified among the sand blasting workers, although no significant differences in RCS were observed between the different processes.

Table 3 shows the associations between climatic, labor-scale, and process variables, and respirable dust levels based on data from Factories A–C. All climatic, process, and labor-scale variables were associated with base-10 logarithmically transformed levels of respirable dust. This multivariate linear regression model had a high predictive capacity (R^2^ = 0.75; adjusted R^2^ = 0.64), and the two strongest predictors were large- versus small-scale labor (effect estimate = 1.35 ± 0.36, *p* = 0.001) and modeling versus office (effect estimate = 0.90 ± 0.25, *p* = 0.002). All predictors in the regression model had VIF values less than 10, indicating no collinear relationships. The DW value was higher than 2.25 (based on the significance level = 0.05, *n* = 30, and nine variables in the model), suggesting no inter-variable correlations of these predictors. The results of residual diagnostics showed that all assumptions in the linear regression analysis were met by this model (Figure S1).

The associations between climatic, labor-scale, and process variables and RCS levels based on data from Factories A–C are shown in Table 4. Only wind speed and process variables were associated with base-10 logarithmically transformed RCS levels. This multivariate linear regression model had a high predictive capacity (R^2^ = 0.89; adjusted R^2^ = 0.84), and the process variables of sand blasting versus office (effect estimate = 2.55 ± 0.38, *p* < 0.001) and sand-box cleaning versus office (effect estimate = 1.75 ± 0.37, *p* < 0.001) were the two strongest predictors in the regression. All variables in the predictive model had VIF values <10, indicating no collinear relationship. These predictors had no inter-variable correlations due to DW values being higher than 2.16 (based on the significance level = 0.05, *n* = 20, and six variables) in the model. All assumptions in the linear regression analysis were met by this model based on the results of residual diagnostics (Figure S2).

Table 5 shows the differences between measured and predicted levels of respirable dust and RCS for different processes at Factory D for the two models. The bias and precision of the predictive model was 0.20 ± 0.60 mg/m^3^ (an accuracy of 0.64 mg/m^3^) for respirable dust and 0.19 ± 0.45 mg/m^3^ (an accuracy of 0.49 mg/m^3^) for RCS, respectively.

**Figure 2:**
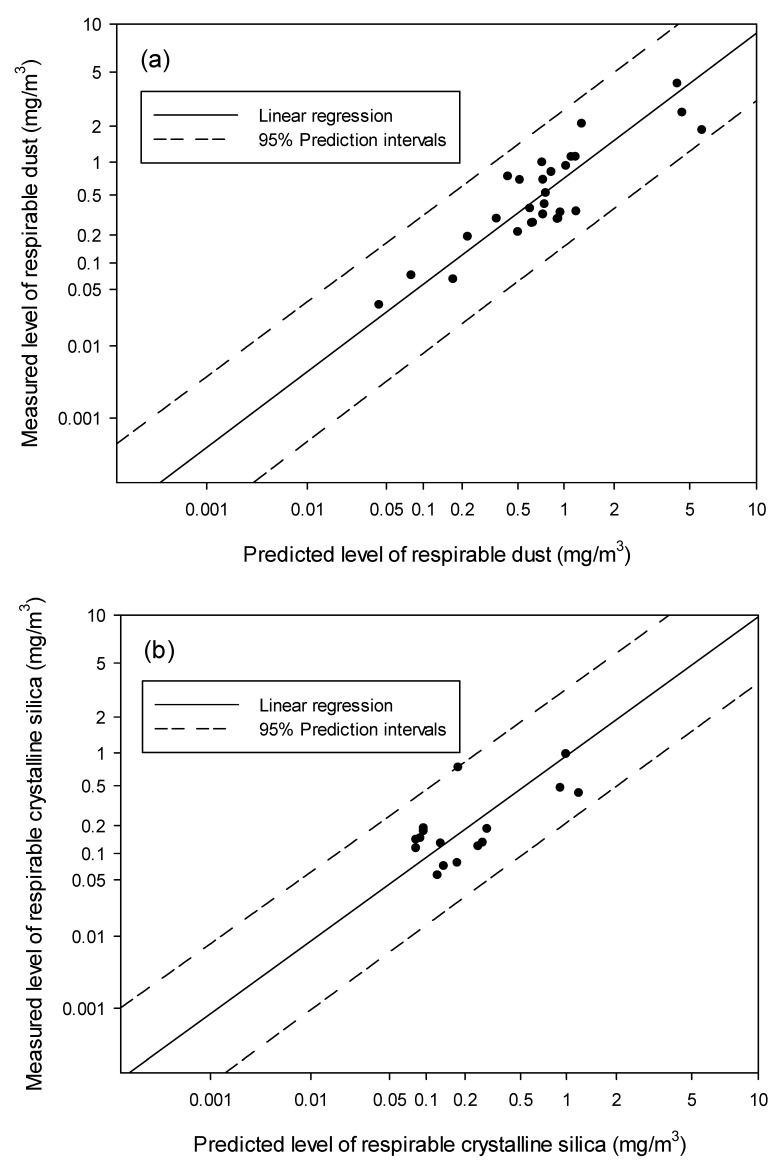
Comparisons of predicted and measured levels of (**a**) respirable dust (*n* = 30) and (**b**) respirable crystalline silica (*n* = 17) above the limit of detection among workers from Factories A–C.

The GM and GSD values of respirable dust (*n* = 38) and RCS (*n* = 19) for measured values above the LOD for Factories A–D were 0.53 ± 3.8 mg/m^3^ and 0.23 ± 3.7 mg/m^3^, respectively. Comparisons between predicted and measured levels of respirable dust and RCS for Factories A–C are shown in Figure 2. The solid line represents the linear regression between predicted and measured levels. All results were located within the 95% prediction intervals (dashed lines).

## 4. Discussion

### 4.1. Main Findings

This study identified that, in Taiwan, foundry workers in the sand blasting process had the highest levels of both respirable dust and RCS. Tossavainen and Kokko [16] reported that fettling (grinding) workers were exposed to the highest mean level (19.8 mg/m^3^) of the total dust of 110 foundry workers in Finland. Furthermore, Yassin et al. [18] observed that spruers (for casting) in the gray iron foundry industry had the highest mean level (0.23 mg/m^3^) of crystalline silica exposure in the United States while in Swedish iron foundries, furnace and ladle repair operators were exposed to the highest mean levels of both respirable dust (1.8 mg/m^3^) and respirable quartz (0.13 mg/m^3^) [19]. These comparisons highlight the degree of variation in both respirable dust and RCS between different types of foundries and the higher levels of respirable dust and RCS among Taiwanese foundry workers as compared to other countries.

The present study illustrates the use of easily collected and accessible information to predict personal levels of respirable dust and RCS with a high degree of accuracy. To the best of our knowledge, this is the first study to establish predictive models for personal exposure to both respirable dust and RCS among foundry workers. These two models could be applied to predict personal exposure for foundry operations in Taiwan which lack sufficient resources to assess exposure. The accuracy of the predictive models for respirable dust (i.e., 0.64 mg/m^3^) below the Taiwan PEL for nuisance dust (5 mg/m^3^), and for RCS (i.e., 0.49 mg/m^3^) below the PEL for type II dust (i.e., 1 mg/m^3^), and between the PEL for type I dust (0.83–0.10 mg/m^3^ for 10–98% crystalline silica), suggest that models are adequate to estimate potential exposure levels during preliminary workplace surveys.

We also identified climatic variables, employee numbers, and process variables as positive and significant factors to predict levels of respirable dust. Previous research has reported that coal miners exposed to high percentages of RCS experienced higher heat stress as compared to those with the lower exposure [36]. Moreover, increased wind speed was found to reduce the RCS levels in construction workers using hand-held grinders [37], and the respirable quartz level above 50 μg/m^3^ was associated with decreased humidity during measurements of farming operations [38]. These findings may indicate different associations between determinants to predict levels of respirable dust and RCS in various industries due to differences in environmental characteristics. The positive association between climatic variables and respirable-dust levels in this study may be attributed to operations requiring higher furnace temperatures and greater volumes of sand to make larger castings, thus generating higher temperatures and exposure levels in the workplace. Fans and water sprinklers are used operationally to decrease temperature and dust levels, leading to increased wind speed and relative humidity. Furthermore, the large-scale labor associated with increased respirable-dust levels in the field might reflect greater production levels and hence the generation of more respirable dust.

Wind speed and process variables were found to be associated with predictive RCS levels. This implies that exposure to RCS was only related to those tasks and working areas where silica sand was used in the manufacturing process. Higher wind speed in the workplace might increase the diffusion of RCS from higher-level work areas to other low-level areas.

### 4.2. Applications

The current eight-hourly averaged PEL of OSHA for RCS is established as 0.05 mg/m^3^ to reduce the risk of silicosis, lung cancer, other lung diseases, and kidney disease [10,11]. Most workers (97%, *n* = 37) at the four factories were exposed to respirable dust below the Taiwan PEL for nuisance dust (5 mg/m^3^). Among 19 RCS samples above the LOD collected from four factories, five out of 18 (28%) measurements were type I dust that was higher than the Taiwan PEL, and the only sample that belonged to type II dust was less than the Taiwan PEL (1 mg/m^3^). However, all 19 RCS samples exceeded the PEL of OSHA, and 16 (84%) were higher than the occupational limit (0.10 mg/m^3^) proposed by the European Commission ACSH [14]. The GM of 19 RCS samples among four factories sampled was 0.23 mg/m^3^; higher than the 0.07 mg/m^3^ found in gray iron foundries in the United States [18] and 0.03 mg/m^3^ found in Swedish iron foundries [19]. In comparison with the Taiwanese and OSHA PELs, adequate control measures (such as isolation and local exhaust ventilation), sound hygiene practices, and personal respirators are necessary to decrease the RCS levels to prevent health hazards among these workers.

This study used the statistical approach (i.e., a stochastic model) rather than knowledge of the physical and chemical mechanisms (i.e., a deterministic model) to predict the personal exposure levels of respirable dust and RCS. Most respirable dust and RCS have the aerodynamic equivalent diameter of less than 10 μm with a 50% cut-point of 4 μm. Recently, the Mastersizer system has been developed to predict different sizes of number concentration of particles by using an optical unit (including a laser beam) to capture the actual scattering pattern from a field of particles. Our established model can predict the component of respirable dust with crystalline silica but cannot separate the RCS into different sizes. Because the smaller particles, such as particulate matter with the aerodynamic equivalent diameter less than 2.5 μm (PM_2.5_), are reported to have the more serious effects on lung cancer [39,40], the mastersizer system can be further applied to our predictive models in future experiments.

In addition, the presented models are limited to the scope of the participating companies. The known but also unknown characteristics in these workplaces may contribute to the established models. For instance, Figure 2b illustrates the limitation of the dataset for RCS that the data form a cluster around 0.2 mg/m^3^ and only four points are apart from this cluster. This observation may come from the limited number of workplace conditions identified in the present study.

### 4.3. Strengths and Limitations

This study is strengthened by representative sampling from different types of foundry companies, detailed information collection, and a comprehensive assessment of exposure to respirable dust and RCS. Furthermore, the predictive models for respirable dust and RCS are established statistically and logically with a high predictive capacity. These models can be used to evaluate potential exposure levels among foundry workers in companies with less than 200 employees.

Notwithstanding, the present study also has some limitations. First, the limited number of cooperating companies and workers resulted in a relatively small sample size. Second, personal sampling on one working day may show variations in exposure due to varied production during busy and slow months. Third, predictive capacity in both respirable dust and RCS is limited to relatively small facilities. Fourth, factors which may affect personal exposure to respirable dust and RCS, such as local ventilation, usage of respirators, and job changes over time, lead to uncertainty when predicting individual exposure levels. Fifth, the percentage of silica in the sand for the RCS model was not considered because 10 of the 27 personal samples were less than the LOD for quartz, cristobalite, and tridymite. Sixth, models did not consider day-to-day variability in exposure assessment. Seventh, models did not account for ventilation that is critical to predicting dust concentrations; and finally, the components of crystalline-free silica were not detected in half of the personal samples (19 out of 38), reducing the predictive capacity of the model.

## 5. Conclusions

This study reported the results of respirable dust and RCS exposure among foundry workers in Taiwan. Generally, most subjects (97%) were exposed to respirable dust levels below the Taiwan PEL, and five out of 38 workers (13%) were exposed to RCS levels higher than the Taiwan PEL. However, all RCS measurements exceeded the OSHA occupational exposure limit. Adequate control measures must be used to reduce the exposure of foundry workers to RCS in Taiwan. Even with some limitations, the designated models demonstrated the acceptable capacity and accuracy to evaluate possible exposure levels among workers in small-scale foundries for further epidemiological studies.

## Figures and Tables

**Table 1:** Descriptive statistical parameters of respirable dust, respirable crystalline silica (RCS), and climatic factors at different companies.

Factory	Number	Respirable Dust Levels (mg/m^3^)				Respirable Crystalline Silica Level (mg/m^3^)				Wind Speed (m/s)		Temperature (°C)		Relative humidity (%)	
		Median	Range	GM	GSD	Median	Range	GM	GSD	Median	Range	Median	Range	Median	Range
A	11	0.35	0.033–0.99	0.30	2.6	0.0015	0.0015–0.42	0.013	12	0.40	0.010–1.1	21 ^b^	20–21	68 ^b^	65–69
B	13	0.52	0.065–51	0.83	5.6	0.13	0.0015–21	0.092	16	0.62	0.080–2.3	16	15–20	51	49–64
C	6	0.87	0.073–1.1	0.52	3.0	0.0015	0.0015–0.17	0.0070	11	0.39	0.020–1.0	24 ^b^	22–27	55	51–66
Total	30	0.39	0.033–51	0.52	4.0	0.075	0.0015–21	0.027	15	0.48	0.010–2.3	21	15–27	59	49–69
*p*-value		0.272 ^a^				0.109 ^a^				0.366 ^a^		<0.001 ^a^		<0.001 ^a^	

GM, geometric mean; GSD, geometric standard deviation. ^a^ Kruskal–Wallis test of the difference between the three groups. ^b^ The significant difference (*p* < 0.05) compared with Factory B (reference) was identified by using the Wilcoxon rank sum test.

**Table 2:** Descriptive statistical parameters of respirable dust, respirable crystalline silica (RCS), and climatic factors for different processes.

Factory	No.	Respirable Dust Levels (mg/m^3^)				Respirable Crystalline Silica Level (mg/m^3^)				Wind Speed (m/s)		Temperature (°C)		Relative Humidity (%)	
		Median	Range	GM	GSD	Median	Range	GM	GSD	Median	Range	Median	Range	Median	Range
Office area	3	0.065	0.033–0.073	0.054	1.5	0.0015	0.0015–0.0015	0.0015	1.0	0.20	0.010–0.080	20 ^b^	19–22	65 ^b^	51–66
Modeling	8	0.35 ^b^	0.21–1.1	0.43	1.8	0.096	0.0015–0.19	0.063	4.8	0.23 ^b^	0.090–0.68	19	16–27	51	50–67
Casting	5	0.29 ^b^	0.19–0.92	0.34	1.8	0.0015	0.0015–0.13	0.0037	7.3	0.82 ^b^	0.13–1.5	21	15–25	58	53–68
Sand-box cleaning	4	0.67	0.41–2.5	0.81	2.3	0.13	0.0015–0.74	0.065	14	0.76	0.39–0.95	21	17–27	55	49–68
Sand blasting	5	0.99 ^b^	0.29–51	1.6	8.1	0.42	0.0015–21	0.099	62	0.67 ^b^	0.39–1.6	20 ^b^	19–25	64	56–69
Polishing	5	1.1 ^b^	0.12–4.2	0.96	3.8	0.15	0.0015–0.97	0.036	20	1.1 ^b^	0.37–2.3	21	15–25	54	51–68
*p*-value		0.026 ^a^				0.164 ^a^				0.009 ^a^		0.970 ^a^		0.425 ^a^	

GM, geometric mean; GSD, geometric standard deviation; No., number. ^a^ Kruskal–Wallis test of the difference between the three groups. ^b^ The significant difference (*p* < 0.05) compared with the office area (reference) was identified by using the Wilcoxon rank sum test.

**Table 3:** Associations between climatic factors, labor scales, production areas, and levels of respirable dust (*n* = 30).

Model	Model 1 ^a^			Model 2 ^b,c^			
Variable	PE	SD	*p*-Value	PE	SD	*p*-Value	VIF Value
Intercept	NA	NA	NA	−7.06	1.69	<0.001	0
Wind speed (m/s)	0.65	0.18	0.001	0.48	0.19	0.019	2.237
Temperature (°C)	−0.005	0.03	0.890	0.15	0.04	0.001	4.692
Relative humidity (%)	−0.01	0.02	0.592	0.04	0.02	0.031	2.975
Large- vs. small-scale labor	0.36	0.22	0.108	1.35	0.36	0.001	7.288
Modeling vs. Office	0.90	0.33	0.012	0.90	0.25	0.002	2.885
Casting vs. office	0.80	0.36	0.035	0.59	0.30	0.061	2.798
Sand-box cleaning vs. office	1.18	0.38	0.004	0.64	0.31	0.051	2.551
Sand blasting vs. office	1.47	0.36	0.001	0.84	0.31	0.013	3.089
Polishing vs. office	1.25	0.36	0.002	0.88	0.33	0.017	3.595
R square				0.75			
Adjusted R square				0.64			
Durbin-Watson value				2.866			
AIC value				−53.37			

AIC, Akaike information criteria; PE, parameter estimate; NA, not available; SD, standard deviation; VIF, variance inflation factor. ^a^ Simple linear regression models for each variable and respirable dust. ^b^ Multiple linear regression adjusted for all variables. ^c^ Multiple linear regression for the final model.

**Table 4:** Associations between climatic factors, labor scales, production areas, and levels of respirable crystalline silica (*n* = 20).

Model	Model 1 ^a^			Model 2 ^b^				Model 3 ^c^			
Variable	PE	SD	*p*-Value	PE	SD	*p*-Value	VIF Value	PE	SD	*p*-Value	VIF Value
Intercept	NA	NA	NA	−8.84	1.74	<0.001	0	−2.84	0.23	<0.001	0
Wind speed (m/s)	1.07	0.30	0.002	0.74	0.20	0.004	3.097	0.55	0.23	0.033	2.260
Temperature (°C)	−0.04	0.07	0.613	0.13	0.04	0.010	3.551				
Relative humidity (%)	0.01	0.03	0.892	0.05	0.02	0.030	4.975				
Large- vs. small-scale labor	0.54	0.43	0.233	1.06	0.35	0.013	7.156				
Modeling vs. Office	1.86	0.31	<0.001	1.87	0.22	<0.001	2.440	1.71	0.28	<0.001	2.283
Casting vs. office	1.93	0.52	0.002	1.20	0.43	0.019	2.021	1.40	0.51	0.016	1.576
Sand-box cleaning vs. office	2.18	0.37	<0.001	1.49	0.29	<0.001	2.421	1.75	0.37	<0.001	2.238
Sand blasting vs. office	3.03	0.37	<0.001	1.94	0.35	<0.001	3.572	2.55	0.38	<0.001	2.378
Polishing vs. office	2.30	0.37	<0.001	1.61	0.34	<0.001	3.338	1.59	0.43	0.003	3.124
R square				0.95				0.89			
Adjusted R square				0.91				0.84			
Durbin-Watson value				2.424				2.749			
AIC value				−42.840				−31.987			

AIC, Akaike information criteria; PE, parameter estimate; NA, not available; SD, standard deviation; VIF, variance inflation factor. ^a^ Simple linear regression models for each variable and respirable crystalline silica. ^b^ Multiple linear regression adjusted for all variables. ^c^ Multiple linear regression for the final model.

**Table 5:** Differences between predictive and measured levels of respirable dust and respirable crystalline silica, for different processes.

Process	Number	Measured Level (mg/m^3^)		Wind Speed (m/s)	Temp (°C)	RH (%)	Predictive Level (mg/m^3^)		Difference (mg/m^3^) ^c^	
		RD	RCS				RD ^b^	RCS ^c^	RD	RCS
Modeling A	1	1.006	0.239	0.46	24.6	62.2	1.728	0.133	0.723	−0.017
Modeling B	1	0.547	0.002 ^a^	0.16	25.3	61.3	1.454	0.091	0.907	0.089
Casting A	1	0.261	0.002 ^a^	0.42	22.0	63.2	0.362	0.062	0.101	0.060
Casting B	1	0.252	0.002 ^a^	0.50	22.0	64.2	0.433	0.068	0.181	0.067
Sand-box cleaning	1	0.647	0.002 ^a^	0.49	24.8	60.7	0.921	0.151	0.274	0.150
Sand blasting	1	3.443	0.002 ^a^	0.73	24.7	63.3	2.324	1.293	−1.119	1.291
Polishing	1	1.778	0.154	0.73	24.4	62.3	2.095	0.142	0.317	−0.012
Office	1	0.066	0.002 ^a^	0.00	26.2	64.2	0.273	0.001	0.207	−0.001
Total	8	1.00 ± 1.13	0.05 ± 0.09	0.44 ± 0.25	24.3 ± 1.5	62.7 ± 1.3	1.20 ± 0.81	0.24 ± 0.43	0.20 ± 0.60	0.19 ± 0.45

RD, respirable dust; RCS, respirable crystalline silica; RH, relative humidity; Temp, temperature. ^a^ Replacement with the half limit of detection (i.e., 0.0015 mg/m^3^). ^b^ Log_10_(RD) = −7.06 + 0.48 × wind speed + 0.15 × temperature + 0.04 × relative humidity + 1.35 × (large- vs. small-scale labor) + 0.90 × (modeling vs. office) + 0.59 × (casting vs. office) + 0.64 × (sand-box cleaning vs. office) + 0.84 × (sand blasting vs. office) + 0.88 × (polishing vs. office). ^c^ Log_10_(RCS) = −2.84 + 0.55 × wind speed + 1.71 × (modeling vs. office) + 1.40 × (casting vs. office) + 1.75 × (sand-box cleaning vs. office) + 2.55 × (sand blasting vs. office) + 1.59 × (polishing vs. office).

## Supplementary Materials

The following are available online at http://www.mdpi.com/1660-4601/15/10/2105/s1, Figure S1: Residual diagnostics of the predictive regression model for base-10 logarithmically translated levels of respirable dust, Figure S2: Residual diagnostics of the predictive regression model for base-10 logarithmically translated levels of respirable crystalline silica.

## Abbreviations

ACGIH	American Conference of Governmental Industrial Hygienists
ACSH	Advisory Committee for Safety and Health at work
DW	Durbin–Watson
GM	geometric mean
GSD	geometric standard deviation
IARC	International Agency for Research on Cancer
ILOSH	Institute of Labor, Occupational Safety and Health
JIS	Japanese Industrial Standards
LOD	limit of detection
NIOSH	National Institute for Occupational Safety and Health
OSHA	Occupational Safety and Health Administration
PE	parameter estimate
PEL	permissible exposure limit
RCS	respirable crystalline silica
SD	standard deviation
SEG	similar exposure groups
TACI	Taiwan Association of Casting Industry
VIF	variance inflation factor
