# Supplementary material for: Determination and Prediction of Respirable Dust and Crystalline-Free Silica in the Taiwanese Foundry Industry
Source: Int J Environ Res Public Health. 2018 Sep 25;15(10):2105. doi: 10.3390/ijerph15102105 (PMC6210212; doi:10.3390/ijerph15102105)

SUPPLEMENTAL MATERIAL

Determination and prediction of respirable dust and crystalline-free silica in the Taiwanese foundry industry

Ching-Tang Kuo[1], Fen-Fen Chiu[2], Bo-Ying Bao[3,4], Ta-Yuan Chang[2,*]

[1] Department of Public Health, College of Public Health, China Medical University, No. 91, Hsueh-Shih Road, Taichung 40402, Taiwan; ctkuo228@gmail.com

[2] Department of Occupational Safety and Health, College of Public Health, China Medical University, No. 91, Hsueh-Shih Road, Taichung 40402, Taiwan; jessica.cy@msa.hinet.net(F.-F.C.); tychang@mail.cmu.edu.tw(T.-Y.C.)

[3] Department of Pharmacy, College of Pharmacy, China Medical University, Taichung, Taiwan. Department of Pharmacy, College of Pharmacy, China Medical University, No. 91, Hsueh-Shih Road, Taichung 40402, Taiwan; bao@mail.cmu.edu.tw

[4] Department of Nursing, Asia University, No. 500, Lioufeng Road, Wufeng, Taichung 41354, Taiwan

* Correspondence: tychang@mail.cmu.edu.tw; Tel.: +886-4-22053366 ext. 6203. Fax: +886-4-22079225.

Figure of contents: Page

Figure S 1. Residual diagnostics of the predictive regression model for base-10 logarithmically translated levels of respirable dust...3

Figure S 2. Residual diagnostics of the predictive regression model for base-10 logarithmically translated levels of respirable crystalline silica 4

Figure S 1. Residual diagnostics of the predictive regression model for base-10 logarithmically translated levels of respirable dust

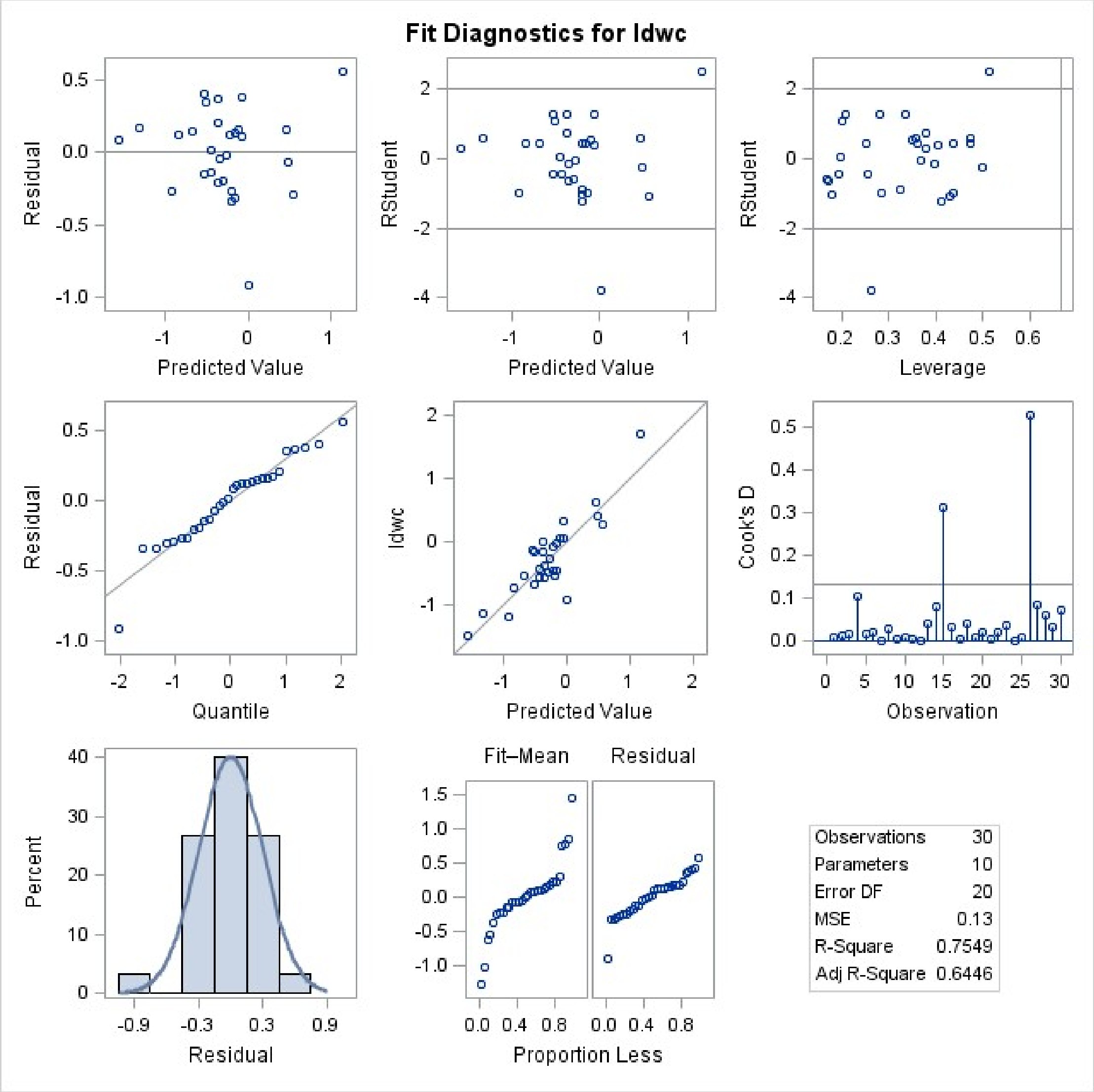

Figure S 2. Residual diagnostics of the predictive regression model for base-10 logarithmically translated levels of respirable crystalline silica

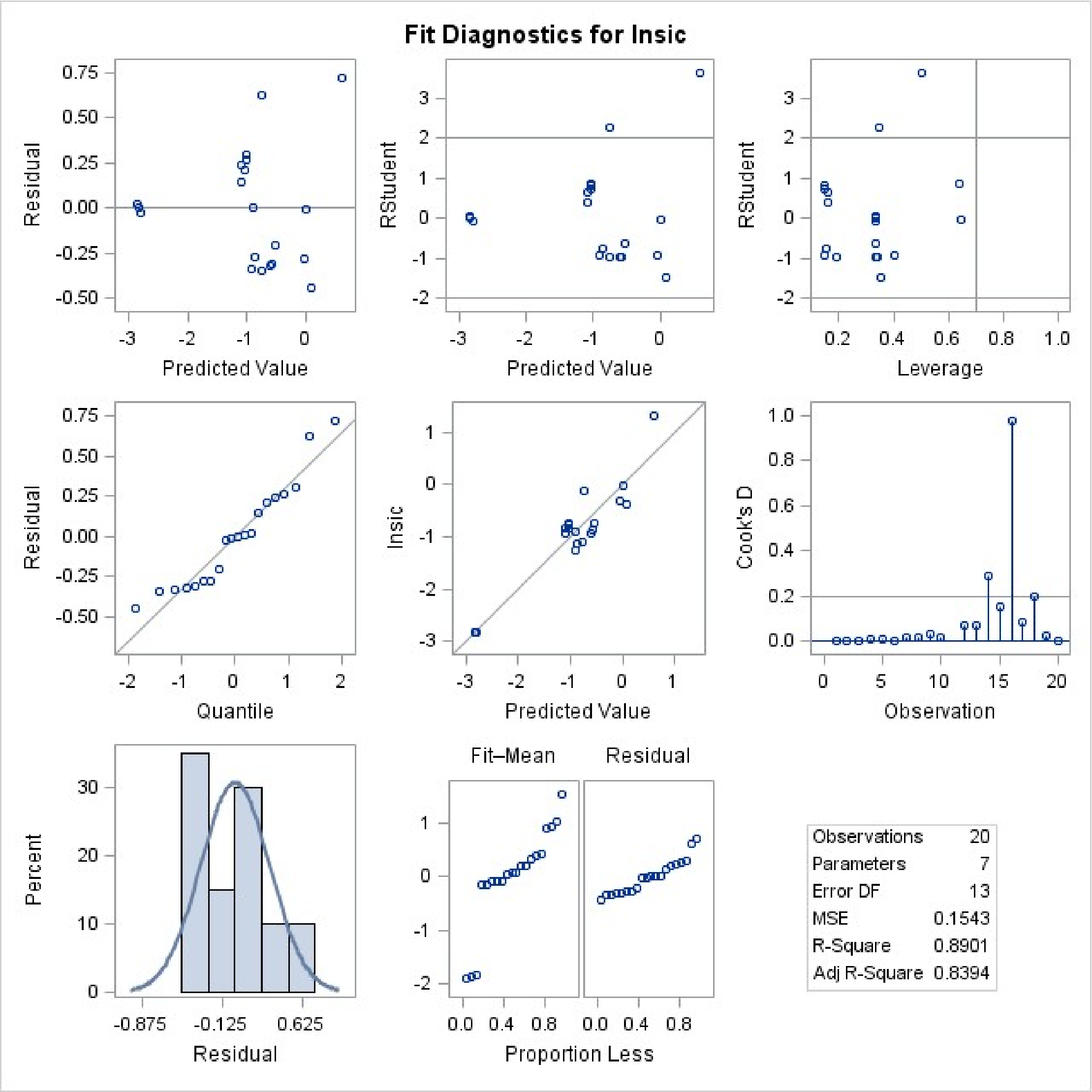

Supplement: Supplementary file 1 [file ijerph-15-02105-s001.pdf]
